# A Segmentation-Assisted Three-Dimensional Planning Workflow for Static-Guided Pterygoid Implant Placement: A Proof-of-Concept Report

**DOI:** 10.3390/jcm15082969

**Published:** 2026-04-14

**Authors:** Andra Patricia David, Silviu Brad, Laura-Cristina Rusu, Ovidiu Tiberiu David, Andra Ardelean, Marius Traian Leretter

**Affiliations:** 1 “Victor Babes” University of Medicine and Pharmacy Timisoara, 2 Eftimie Murgu Sq., 300041 Timisoara, Romania; andra.david@umft.ro (A.P.D.); ardelean.andra@umft.ro (A.A.); 2 Department of Radiology, “Victor Babes” University of Medicine and Pharmacy Timisoara, 2 Eftimie Murgu Sq., 300041 Timisoara, Romania; brad.silviu@umft.ro; 3 Department of Oral Pathology, Multidisciplinary Center for Research, Evaluation, Diagnosis and Therapies in Oral Medicine, “Victor Babes” University of Medicine and Pharmacy Timisoara, 2 Eftimie Murgu Sq., 300041 Timisoara, Romania; 4 Faculty of Physics, West University of Timisoara, 4 Vasile Parvan Blvd., 300223 Timisoara, Romania; 5 Department of Functional Sciences, Multidisciplinary Center for Research, Evaluation, Diagnosis and Therapies in Oral Medicine, “Victor Babes” University of Medicine and Pharmacy Timisoara, 2 Eftimie Murgu Sq., 300041 Timisoara, Romania; 6 Radiologie CBCT SRL, Iulius Mall, 2 Consiliul Europei Sq., 300627 Timisoara, Romania; 7 Department of Prosthodontics, Multidisciplinary Center for Research, Evaluation, Diagnosis and Therapies in Oral Medicine, “Victor Babes” University of Medicine and Pharmacy Timisoara, 2 Eftimie Murgu Sq., 300041 Timisoara, Romania; leretter.marius@umft.ro

**Keywords:** dental implants, pterygoid region, cone-beam computed tomography, surgical guides, three-dimensional imaging, computer-assisted surgery, maxillary atrophy, implant planning

## Abstract

**Background/Objectives**: Pterygoid implant placement represents a valuable alternative to conventional bone grafting procedures in the rehabilitation of the atrophic posterior maxilla; however, the procedure remains technically demanding because of limited visibility, difficult access, complex pterygomaxillary anatomy, and the need for precise angulation and distal bicortical anchorage. Although digital guidance has increasingly been applied in implant dentistry, a clearly described workflow integrating automatic segmentation, selective virtual trimming of the posterior maxillary anatomy, and direct three-dimensional planning for static-guided pterygoid implant placement remains insufficiently detailed in the literature. The aim of this report was to describe and illustrate such a workflow in a proof-of-concept clinical application. **Methods**: This work was designed as a methodological proof-of-concept with a single clinical illustration. A CBCT dataset was imported into BlueSkyPlan, where automatic segmentation was used to generate three-dimensional models of the maxilla, teeth, and pterygoid process. The segmented volumes were then selectively trimmed to expose the relevant pterygomaxillary anatomy and to support direct three-dimensional planning of the implant axis in the rendered model. A static surgical guide with combined tooth and mucosal support was subsequently designed, positioned on a printed jaw model derived from the intraoral scan, and assessed by CBCT-based internal verification. **Results**: In this proof-of-concept application, the workflow enabled three-dimensional visualization of the pterygomaxillary trajectory, supported implant axis planning in the rendered model, and facilitated guide design and radiographic verification of the planned trajectory. The verification step provided an internal methodological consistency check between the planned implant axis and the drill-guided direction visible on CBCT. **Conclusions**: The present report describes a segmentation-assisted three-dimensional planning workflow for static-guided pterygoid implant placement in a single proof-of-concept clinical application. The workflow should be interpreted as a methodological illustration rather than a quantitative validation study. Further investigations are required to evaluate accuracy, inter-operator reproducibility, and broader clinical applicability.

## 1. Introduction

Pterygoid implants have become an established alternative to bone grafting procedures in selected cases of posterior maxillary atrophy [1,2,3], especially when distal anchorage is required and when sinus augmentation, short implants, cantilever extension, or more invasive reconstructive approaches are considered less favorable [4]. Systematic reviews and long-term clinical reports have consistently supported the clinical viability of this treatment concept [5,6,7], while also emphasizing that placement in this region remains one of the most operator-sensitive procedures in implant dentistry [8]. Current evidence suggests that these implants can achieve high survival rates, supporting their use in the rehabilitation of the atrophic posterior maxilla [9]. The technical difficulty of this procedure is related to limited surgical access [10], constrained visualization of the distal osteotomy path [11], the oblique angulation required to reach the pterygomaxillary region, and the morphological variability of the posterior maxilla [12]. In this region, three bones—the maxillary tuberosity, the pterygoid process of the sphenoid bone, and the pyramidal process of the palatine bone—form a symphysis zone that can be used for anchorage and may provide superior primary stabilization [3,13]. Tomographic analyses of the atrophic maxilla have further emphasized the importance of preoperative assessment of bone availability and surgical safety in the pterygomaxillary region [14]. Variations involving the greater palatine canal should be considered to reduce the risk of neurovascular injury in posterior maxillary surgery [15]. Posterior maxillary configuration may influence the course of neurovascular structures, which is relevant when selecting a safe implant trajectory [16]. Morphological studies of the pyramidal process and adjacent cortical structures help explain why cortical engagement is critical for implant stability in this area [17]. CBCT-based anatomical studies have shown that bone availability, density, sinus relationships, palatal morphology, and the spatial configuration of the pterygomaxillary corridor vary substantially among patients [9,18]. For this reason, pterygoid implant placement should not be regarded as a simple distal extension of conventional implant placement, but rather as a distinct anatomical and prosthetically driven procedure that requires careful three-dimensional planning [19]. In severely atrophic maxillae, pterygoid implants should also be considered in relation to zygomatic implants, which represent another option used to reduce reliance on more extensive grafting procedures in posterior maxillary rehabilitation. Although both approaches may reduce the need for sinus augmentation and extensive bone grafting, they are not fully interchangeable. Zygomatic implants are generally considered in more advanced maxillary resorption and are often integrated into full-arch rehabilitation concepts, whereas pterygoid implants may provide a less invasive distal anchorage strategy in selected cases with residual posterior maxillary support and favorable pterygomaxillary anatomy. Therefore, the choice between these approaches remains anatomy-dependent, prosthetically driven, and clinician-specific. Recent evidence confirms favorable outcomes for pterygoid implants, while also highlighting the need for more standardized and operator-independent workflows [6]. In parallel with the increased use of digital dentistry, several authors have explored computer-assisted approaches for pterygoid implant placement, including image-based customized drill guides, static templates, dynamic navigation systems, and, more recently, robot-assisted protocols [20]. These studies suggest that guided surgery may improve trajectory control and standardization, even if survival rates alone do not always demonstrate a clear superiority over experienced freehand surgery [21]. In reality, the potential advantage of digital guidance in the pterygoid region is not limited to implant survival. It also concerns control over angulation, emergence, avoidance of undesirable sinus intrusion, and reduction in technique sensitivity [22]. Biomechanical studies suggest that implant angulation and cortical engagement may significantly influence stress distribution, and an optimal pterygoid approach has not yet been universally defined [23]. Nevertheless, currently available digital methods still leave several practical issues insufficiently resolved in daily clinical work. In many software environments, the operator continues to depend heavily on multiplanar slices for trajectory definition, while the three-dimensional rendered scene is underused as an actual planning workspace. In addition, not all protocols make effective use of automated segmentation and selective trimming of the segmented anatomy in order to expose the true implant corridor and to simplify guide design in the posterior maxilla. As highlighted in our previous *Medicina* article on mini-implant planning, automatic segmentation can transform the CBCT dataset into a practical three-dimensional study model and can shift planning from a predominantly sectional analysis toward direct spatial reasoning [24]. Despite the growing use of digitally assisted implant planning, the literature still lacks a clearly described and transferable workflow for static-guided pterygoid implant placement in which automatic CBCT segmentation, selective virtual trimming of the posterior maxillary anatomy, and direct three-dimensional spatial planning are integrated into a single planning logic. In many currently described approaches, the rendered three-dimensional model remains largely illustrative rather than functioning as the primary planning workspace. This limitation is particularly relevant in the pterygomaxillary region, where the implant trajectory is long, oblique, anatomically variable, and difficult to conceptualize using sectional analysis alone. In this context, the present work addresses a methodological gap by describing a proof-of-concept workflow in which segmentation is used not merely for visualization, but as a planning-support tool that simplifies the distal corridor, facilitates direct spatial reasoning, and supports the design of a static surgical guide for pterygoid implant transfer. BlueSkyPlan was used in the present study because, beyond its applicability to pterygoid implant planning, it is an open and easily accessible system that allows free segmentation, thus supporting reproducibility of the proposed workflow by a broader clinical and research community. Whereas our previous work addressed segmentation-assisted planning in the context of orthodontic mini-implant insertion, the present manuscript extends the same conceptual shift toward direct three-dimensional spatial planning to a substantially more complex posterior maxillary implant trajectory. The objective of the present manuscript was therefore not to validate a finalized protocol, but to describe and clinically illustrate a methodological proof-of-concept workflow for segmentation-assisted static-guided pterygoid implant planning that may support future quantitative validation studies.

## 2. Materials and Methods

### 2.1. Study Design and Positioning of the Manuscript

This work was designed as a methodological proof-of-concept with a single clinical illustration. The workflow, rather than the patient, served as the innovation unit. The present manuscript was not intended as a comparative, controlled, or quantitative validation study, but rather as a structured description of the workflow logic and its implementation in one clinically relevant case. The proof-of-concept component was included to demonstrate practicality in real-world clinical settings, including image acquisition, segmentation, implant planning, guide creation, guide placement, and intraoperative use. The case was selected as a clinically relevant proof-of-concept example of posterior maxillary rehabilitation requiring pterygoid trajectory planning. No claim is made that this case is representative of the full range of anatomical scenarios, and the use of a single case does not permit generalization regarding accuracy, reproducibility, or broader clinical applicability. The patient was treated in ordinary clinical practice following conventional diagnostic examinations, informed consent for treatment, and anonymous scientific use of the records.

### 2.2. Imaging Acquisition

The patient completed a CBCT examination for the maxilla. The CBCT system used was a VATECH Green X (model PHT-75CHS), manufactured by VATECH, Yongin, Republic of Korea, and CBCT images were acquired using a voxel size of 0.2 mm, a tube voltage of 94 kVp, an X-ray tube current of 6 mA, and a field of view of 9 × 12 cm. It was decided to use the 0.2 mm voxel rather than the 0.3 mm voxel in order to improve the resolution of the CBCT image as well as the quality of segmentation.

### 2.3. Software Environment and Segmentation Logic

**Figure 1:**
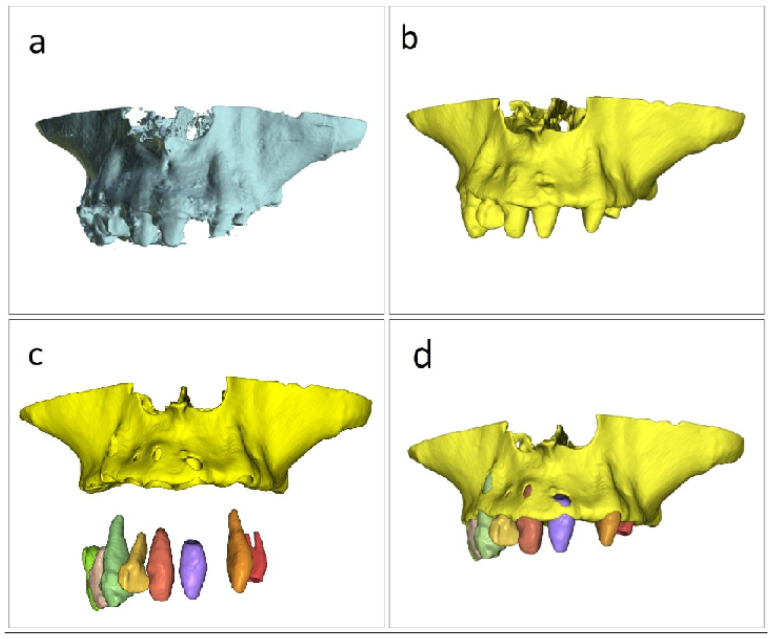
Automatic segmentation workflow in BlueSkyPlan software for converting the CBCT DICOM dataset into a three-dimensional study model. (**a**) Initial 3D DICOM dataset. (**b**) Segmentation with the teeth merged with the jaw, resulting in a single STL file. (**c**) Segmentation with the teeth and jaw generated as separate STL files. (**d**) Final composite fusion model obtained from a single segmentation process that generated multiple segmented components.

The initial stage of the approach was converting the 3D DICOM dataset from Figure 1a into an interactive three-dimensional study model using automatic segmentation. Two types of segmentations can be obtained for use in our study using the workflow in the BlueSkyPlan software version 5.0.29 (64 bit) from Blue Sky Bio LLC, Libertyville, IL, USA: the first, where the teeth are merged with the jaw as shown in Figure 1b, resulting in a single .stl file from the segmentation, and the second, where the teeth and jaw are distinct .stl files, as shown in Figure 1c. In our study, we used multiple segmentations, which combine all parts, including those that are distinct as separate files, into a composed .stl file, as seen in Figure 1d. In the present workflow, automatic segmentation was used as a planning-support tool to generate manipulable three-dimensional models for spatial analysis and guide development. No formal assessment of segmentation thresholds, segmentation accuracy, manual correction effects, or inter-operator variability was performed in this proof-of-concept, and these aspects should be regarded as limitations of the present report.

We chose this type of segmentation since it enables virtual tooth extractions or individual modeling if the working approach requires it. This composed .stl file from Figure 1d is appropriate for determining the pterygoid implant trajectory in relation to the posterior maxillary anatomy. The segmentation is done in the software’s Model Master module, where the Automatic Jaw Segmentation option is selected from the Tool button, keeping the software’s “Merge teeth with jaws” option unchecked. Although the workflow is illustrated here within a specific software environment, the underlying methodological principle is not intended to be software-exclusive, but rather to emphasize the planning logic of segmentation-assisted spatial simplification and direct three-dimensional implant trajectory analysis.

### 2.4. Description of Cutting Plane

**Figure 2:**
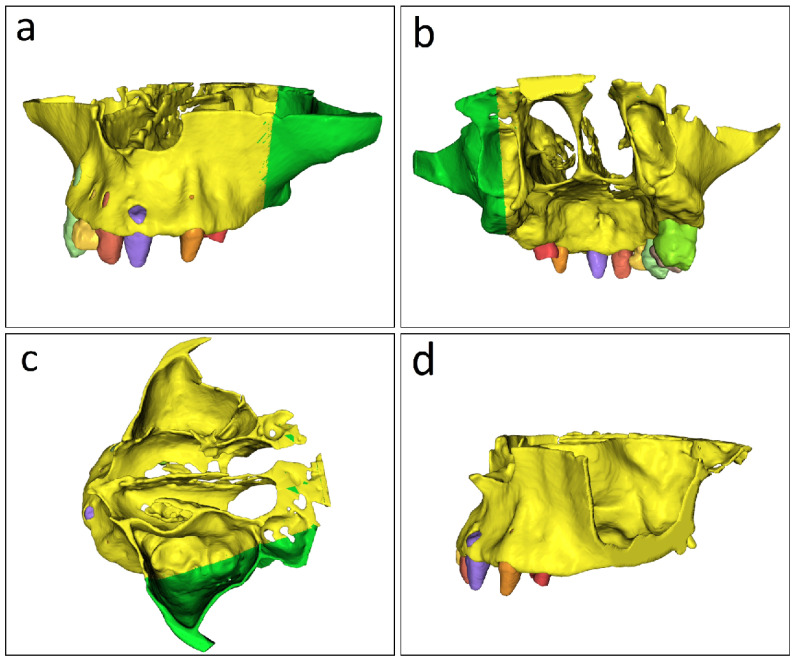
Spatial description of the cutting plane shown in (**d**). (**a**) Positioning of the plane in the maxillary panoramic plane, parallel to the tooth axes. (**b**) Passage of the plane through the center of the maxillary crest. (**c**) Passage of the plane between the medial and lateral laminae of the sphenoid pterygoid process, exiting through the pterygoid fossa. (**d**) Three-dimensional representation of the cutting plane.

The spatial description of the cutting plane from Figure 2d must meet the following conditions:

(a) Located in a maxillary panoramic plane, parallel to the axes of the teeth (Figure 2a).

(b) Passes through the center of the maxillary crest (Figure 2b), and between the two medial and lateral laminae of the sphenoid’s pterygoid process, exiting through the pterygoid fossa (Figure 2c).

**Figure 3:**
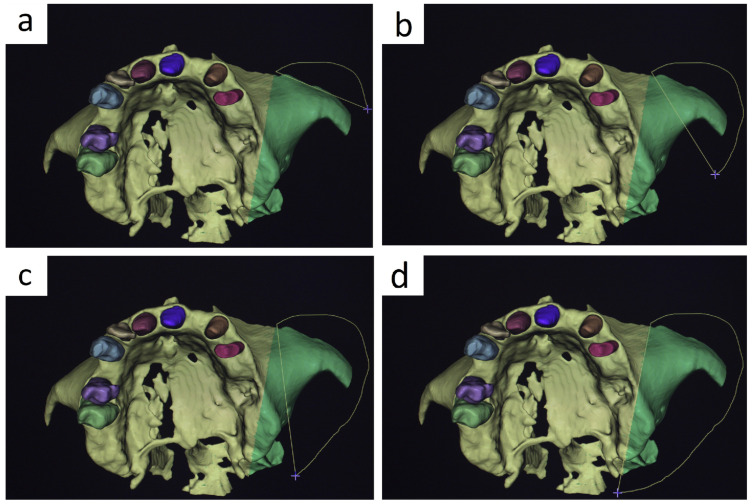
Application of the lasso cutting function to define the contour of the region to be removed and generate a straight cutting plane. (**a**) Starting point of the contour. (**b**,**c**) Intermediate points of contour tracing. (**d**) Endpoint of the contour; together with the starting point, this defines the final cutting plane.

Condition (a) is related to the prosthetic side of the problem, with the implant aligned with the other teeth; condition (b) optimizes the implant’s safety during insertion by maintaining an equal distance from the palatal and vestibular cortices; and condition (c) is related to the implant’s stability, as it passes through the maxillo-pterygoid bicortical structures, as shown in Figure 2a,b. The initial virtual trimming procedure applied to the segmented STL model was guided by the spatial orientation of the rendered anatomy and does not refer to any intraoperative surgical cutting maneuver. Thus, the STL file is rotated in the 3D rendering view window until the occlusal plane is parallel to the PC view window, allowing us to see the contour of the maxillary crest (Figure 3). In this position, it is also required to clearly visualize the sphenoid’s pterygoid process, which is located posterior to the maxillary tuberosity and is made up of the medial and lateral laminas, between which the pterygoid fossa is defined. The lasso cutting function creates an outline of the part we wish to eliminate. To obtain a straight cutting plane, trace a straight line from the contour’s initial and final points. These two positions set the final cutting plane. Figure 3a depicts the contour’s starting point, while Figure 3b,c show the contour’s intermediate points. Figure 3d represents the end of the contour tracing, which contains the part to be cut, and the straight line in this image represents the cutting plane. The software’s CUT feature digitally removes the selected portion of the STL model from the rendered scene. The cutting plane definition should therefore be interpreted as an anatomically guided planning heuristic within the present workflow rather than as a quantitatively validated universal parameter set. Future studies should investigate inter-operator consistency and whether this step can be translated into more reproducible quantitative planning criteria across cases.

**Figure 4:**
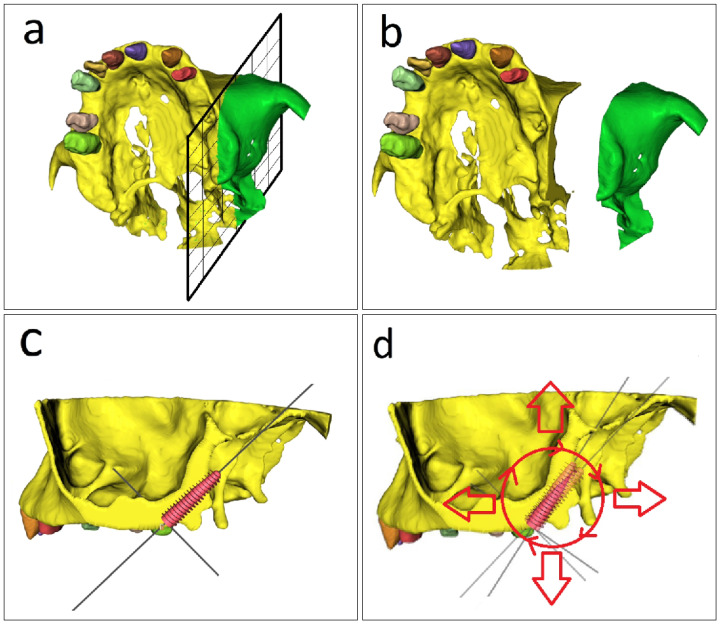
Orientation of the cutting plane and preliminary implant planning. (**a**) Spatially oriented cutting plane providing a descriptive view of the cutting procedure. (**b**) Segmented file separated into two parts, with the green portion indicating the part selected for deletion. (**c**) Remaining model after removal, showing clearer visualization of the maxillary sinus and the posterior margin of the pterygopalatine fossa. (**d**) Final planned implant position, which can be rotated to different angulations or translated to different locations according to the treatment plan and the type of pterygoid implant used.

Figure 4a illustrates the spatially oriented cutting plan, which provides a more complete and descriptive description of the cutting procedure, whereas Figure 4b shows the segmented file separated into two parts, with the green part representing the one to be deleted. Figure 4c shows how the remaining yellow area of the maxillary sinus and the posterior margin of the pterygopalatine fossa are more visible after this is removed.

### 2.5. Planning the Pterygoid Implant

Figure 4d represents the plan for the final location of the implant for insertion, which can be rotated at various inclination angles or translated to different positions based on the treatment plan and the type of pterygoid implant utilized. The predicted spatial direction of the pterygoid implant, also known as the implant’s axis of symmetry, is an imaginary line of orientation along which the implant should be positioned from the maxillary tuberosity area to the pterygomaxillary/pterygoid region. For the proof-of-concept application, a pterygoid implant from the BIO|Pterygoid category (Blue Sky Bio LLC, Libertyville, IL, USA), type IPJN4518, was selected. The implant had an apical diameter of 3.70 mm, an occlusal diameter of 4.50 mm, and a length of 18.00 mm. This implant configuration was considered appropriate for the intended long oblique pterygomaxillary trajectory and for achieving distal bicortical anchorage in the posterior maxilla. In the present workflow, implant selection was integrated with the spatial planning stage, since implant dimensions and macrogeometry directly influenced the final implant axis, sleeve positioning, and guide design. No comparison with alternative implant systems or planning strategies was performed in the present proof-of-concept.

### 2.6. Guide Design and Support Concept

**Figure 5:**
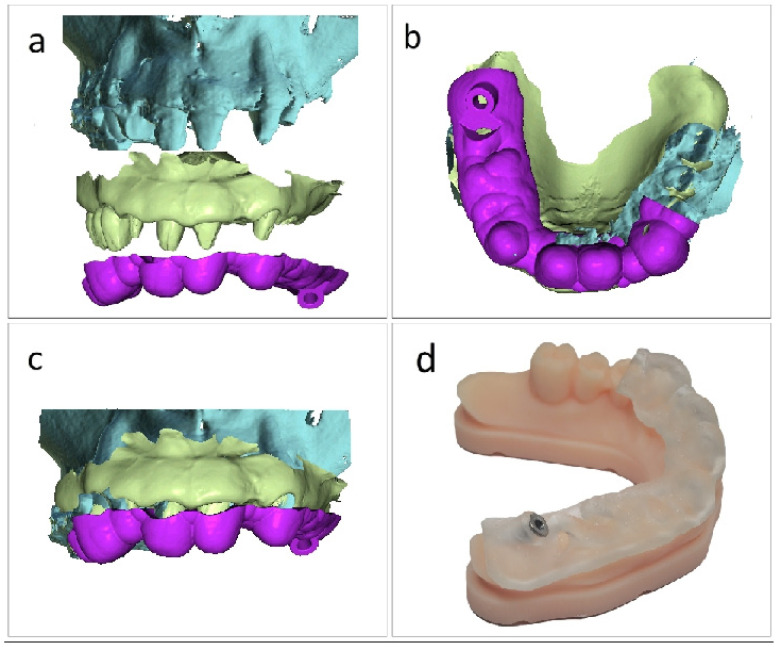
Guide design and support concept. (**a**) DICOM file, intraoral scan, and surgical guide expanded view (**b**) Occlusal view of the crown- and mucosa-supported surgical guide design. (**c**) Rendered validation of intraoral scan superimposition onto the DICOM dataset. (**d**) Final surgical guide with titanium inner sleeve and verification of its stable, unique position on the printed jaw model.

The surgical guide was produced using the BlueSkyPlan software after fitting the implant with the above criteria. Digital impressions were taken with an Aoralscan 3 intraoral scanner (SHINING 3D Dental, Hangzhou, China). The software’s AI feature automatically superimposes the intraoral scan onto the DICOM file during the matching step (Figure 5a). The guide is designed in such a way that it provides support to both the crowns and the mucosa (Figure 5b). The positioning of the intraoral scan over the DICOM file is radiographically validated in the sections of the program, as well as rendering in the superimposition of the files in Figure 5c. The surgical guide used a StecoGuide system (Steco-System-Technik, Hamburg, Germany) and titanium inner sleeve (REF M.27.03.D210L6; outer diameter 3.5 mm, inner diameter 2.1 mm, length 6.0 mm); see Figure 5d.

The design of the guide consists of outlining the area where the guide is to be placed and customizing the support for the metallic sleeve. Then, from the software, the command to create the guide is executed, and the STL file of the guide is obtained, which is sent to the 3D printer. Next, the metal sleeve is inserted into the guide, and the final phase is polymerisation with UV light in the polymerisation oven. The verification of the guide’s placement is then done on the printed model of the jaw obtained from the intraoral scan (Figure 5d). Guide seating was assessed descriptively in the present proof-of-concept application. No quantitative analysis of manufacturing tolerances, sleeve-position deviations, printing distortion, or polymerization-related dimensional changes was performed.

## 3. Results

### 3.1. Workflow Feasibility

In this proof-of-concept application, the workflow enabled three-dimensional visualization of the pterygomaxillary trajectory and supported implant axis planning in the rendered model. Automatic segmentation generated sufficiently usable three-dimensional models of the maxilla and dentition for planning purposes. The segmented structures could then be selectively trimmed to reveal the posterior anatomical region and the pterygoid implant axis trajectory more clearly than would have been possible by relying on the intact volume alone. The observations reported here should be interpreted descriptively, as no quantitative visualization or planning metrics were collected.

### 3.2. Method Verification

**Figure 6:**
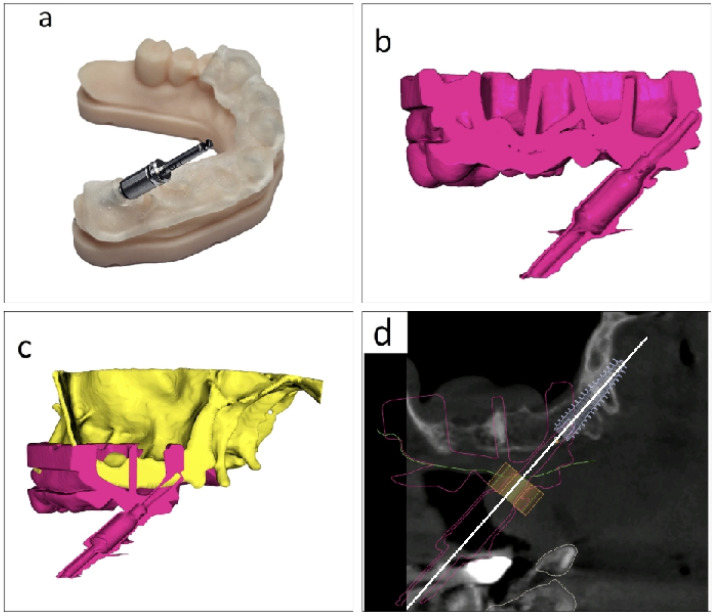
Method verification. (**a**) CBCT scan of the model with the surgical guide in place and a 2.1 mm pterygoid drill inserted through the guide sleeve. (**b**) Sectioned STL model generated from the CBCT scan, showing the internal geometry of the guide–drill assembly along the implant axis. (**c**) Rendering alignment of the sectioned STL model with the patient’s DICOM dataset segmented. (**d**) CBCT radiographic section showing coincidence between the implant axis, marked by a white line, and the axis of the drill inserted through the guide, confirming the accuracy of both the guide and the technique.

To assess the internal methodological consistency of the process applied in this work, we utilized a CBCT scan of the model with the surgical guide overlaid, as seen in Figure 6a, where we inserted a drill equal to the diameter of the sleeve’s aperture, specifically 2.1 mm. The tool utilized was a pterygoid drill (Ø 2.1 mm; Ref. GD-PT-21) from the Pterygo surgical kit (B&B Dental, Bologna, Italy). The idea for utilizing the CBCT scan instead of an optical scan was to acquire data relative to the axis of symmetry of the implant. Splitting provided insights into the interior geometry of this two-piece structure (Figure 6b). After acquisition of the CBCT image using the identical dental tomograph and exposure conditions outlined in Section 2.2, we imported the DICOM images into the BlueSkyPlan software via the sequence Model Master–File–Import DICOMS–Model Scan. The software automatically generates an STL file similar to that shown in Figure 6b, which was sectioned using the identical plane aligned with the implant axis. After that, this file is aligned with the patient’s DICOM radiographic images, as illustrated in Figure 6c,d. In Figure 6d, which represents a CBCT radiographic section, we marked the implant axis with a white line, which coincides with the axis of the drill placed on the guide. This verification step should be interpreted as an internal methodological consistency check rather than as an independent gold-standard accuracy validation, since no external reference system or angular and linear deviation measurement protocol was used.

**Figure 7:**
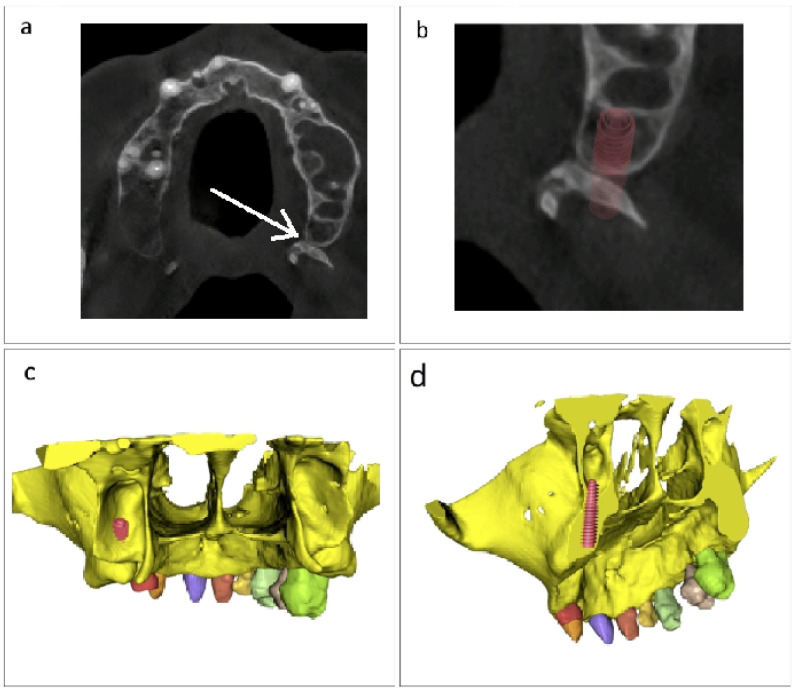
Radiographic confirmation of implant bicorticality and spatial position. (**a**) Axial section obtained before implant placement, with the relevant cortical area indicated by the white arrow. (**b**) Axial section showing the implant position achieved with the proposed method. (**c**) Posterior view of the implant located in the pterygoid fossa, confirming its posterior spatial position. (**d**) Inclined axial section along the implant axis, performed below the lower axis of the implant. The visible overextension in the image corresponds to trajectory visualization for radiographic verification purposes and does not indicate intentional extension beyond the clinically planned implant endpoint.

To radiographically confirm the bicorticality of the implant insertion, we performed two axial sections across the maxillary and pterygoid cortices, the initial section being prior to implant implantation as shown in Figure 7a, with the relevant area marked by the white arrow. The second part in Figure 7b illustrates the location where the implant is positioned with the method from our study. Figure 7c illustrates a posterior view of the implant located in the pterygoid fossa to confirm its posterior spatial position. The final inspection was conducted in an inclined axial plane along the implant axis, except for the section below the lower axis of the implant, as seen in Figure 7d. The apparent apical overextension visible in the image reflects the radiographic visualization of the drill-guided axis used for methodological verification of trajectory alignment and should not be interpreted as intentional clinical overpreparation beyond the planned implant endpoint.

## 4. Discussion

The present report should be interpreted as a method-centered proof-of-concept contribution to the pterygoid implant literature. Previous reviews and cohort studies have already established that pterygoid implants can be clinically effective in the rehabilitation of the atrophic posterior maxilla. What remains less clearly defined is the practical manner in which the distal trajectory is visualized, simplified, and transferred into a guide design that is stable enough for routine use.

Recent literature has explored several digital solutions for this problem. Static-guided pterygoid implant studies, dynamic navigation reports, and robot-assisted workflows all indicate that digital assistance can improve planning control and reduce reliance on purely freehand estimation. However, these modalities differ substantially in cost, equipment requirements, learning curve, and clinical accessibility. The method proposed here is positioned between conventional freehand planning and more technologically demanding navigation or robotic systems. It seeks to preserve the accessibility of a static-guide approach while strengthening the preoperative planning stage through segmentation-assisted spatial reasoning. Our workflow may be compared with recent reports of static-guided pterygoid implant placement using 3D-printed templates, which reported clinically acceptable deviations and procedural predictability [25]. In line with studies comparing dynamic navigation and free-hand surgery, the present proof-of-concept supports the concept that digitally assisted workflows may improve trajectory control in pterygoid implant placement [26]. Previous studies using dynamic navigation for pterygoid implants have similarly emphasized improved visibility, real-time control, and enhanced accuracy in this anatomically demanding region [27]. Although short-term implant success may not differ substantially between guided and non-guided placement, guided workflows may still offer advantages in anatomic safety and prosthetically driven positioning [28]. For comparison, robotic-assisted workflows represent a more advanced form of digital guidance, but the underlying objective remains similar: improved control in a region with difficult access and limited visibility [29]. Although developed for another posterior maxillary application, BlueSkyPlan-based segmentation and customized guide design have shown that digitally assisted posterior workflows can be translated into clinically precise procedures [30]. More broadly, the present method can be interpreted within the context of both static and dynamic computer-aided protocols developed for edentulous maxillary rehabilitation involving pterygoid implants [31]. The originality of the present work lies more narrowly in the integrated sequence of steps: automatic segmentation of the CBCT dataset, conversion into manipulable surface models, targeted trimming of the segmented anatomy to reveal the distal corridor, direct implant simulation in the three-dimensional rendered scene, and fabrication of a mixed-support guide adapted to the posterior maxillary support conditions [30,32]. The concept of this workflow can be traced back to our article in Medicina on automated segmentation for guided insertion of orthodontic mini-implants, in which the central methodological change was the shift in implant simulation from predominantly sectional planning to direct three-dimensional spatial planning based on automatically segmented anatomy [24]. The current pterygoid workflow extends this principle to a more demanding implantological environment, where the challenge is not only to identify a narrow interradicular space, but also to define a long oblique trajectory through a posterior maxilla and a pterygoid process whose anatomy is more complex and less visually intuitive [28]. The pterygomaxillary region is anatomically variable, and the literature has repeatedly stressed the importance of individualized CBCT analysis for determining angulation, length, distal anchorage strategy, and sinus relationship. In this context, the present method offers a practical way to convert anatomical variability from an obstacle into a visible planning parameter. By trimming the segmented volume and exposing the path directly in three-dimensional space, the operator can reason about the intended axis in a more intuitive and clinically transferable manner. Biomechanical analyses suggest that pterygoid implant angulation may substantially influence stress distribution, supporting the rationale for carefully controlled guided trajectories [33]. Alternative orientation methods based on intraoral landmarks have also been proposed, but digitally planned guidance may offer a more reproducible approach in this region [34]. Clinically, the main relevance of the proposed method lies in making the intended trajectory visible, editable, and transferable within a single digital chain. A second clinically relevant point is the mixed-support guide concept. In the posterior maxilla, guide stability is often more nuanced than the simple labels tooth-supported or mucosa-supported suggest. By explicitly incorporating teeth, crowns, and mucosa into the support logic, the proposed workflow acknowledges actual clinical support conditions and may offer better seating behavior in selected cases [32].

Several limitations of the present proof-of-concept should be acknowledged. First, the workflow was illustrated in a single clinical case, which does not permit generalization regarding reproducibility, operator-independence, or broader clinical applicability. Second, no formal quantitative validation was performed, and neither angular nor linear deviations between planned and achieved trajectories were measured. Third, the segmentation step was used as a planning-support tool, but no formal assessment of segmentation accuracy, threshold sensitivity, or inter-operator variability was undertaken. Fourth, the verification procedure was based on internal radiographic alignment within the same digital workflow and should therefore be interpreted as a methodological consistency check rather than as an independent gold-standard accuracy validation. Finally, potential sources of error related to guide fabrication, sleeve positioning, CBCT resolution, printing distortion, and polymerization-related dimensional changes were not quantitatively analyzed. These limitations indicate that the present report should be interpreted as a methodological illustration intended to support future validation studies rather than as definitive evidence of workflow robustness.

## 5. Conclusions

The present report describes a methodological proof-of-concept workflow for segmentation-assisted pterygoid implant planning, guide design, and CBCT-based internal verification in a single clinical application. By combining automatic segmentation, selective virtual trimming, direct three-dimensional spatial planning, and guide development within one digital sequence, the workflow illustrates a possible approach for improving trajectory visualization in the anatomically complex posterior maxilla. However, the findings should be interpreted descriptively and not as evidence of validated reproducibility or quantitative accuracy. Further studies are needed to assess deviation metrics, inter-operator consistency, and broader clinical applicability across different anatomical scenarios.

## Data Availability

Because the study includes patient-derived imaging data, the full datasets are not publicly available for privacy and ethical reasons. De-identified methodological details related to the workflow may be made available from the corresponding authors upon reasonable request, subject to ethical and legal constraints.
